# Artificial light at night, in interaction with spring temperature, modulates timing of reproduction in a passerine bird

**DOI:** 10.1002/eap.2062

**Published:** 2020-01-13

**Authors:** Davide M. Dominoni, Johan Kjellberg Jensen, Maaike de Jong, Marcel E. Visser, Kamiel Spoelstra

**Affiliations:** ^1^ Department of Animal Ecology Netherlands Institute of Ecology (NIOO‐KNAW) Wageningen The Netherlands; ^2^ Institute of Biodiversity, Animal Health and Comparative Medicine University of Glasgow Glasgow G128PG United Kingdom; ^3^ Department of Biology Lund University Lund Sweden; ^4^ Plant Ecology and Nature Conservation Group Wageningen University Wageningen The Netherlands

**Keywords:** artificial light at night, light pollution, *Parus major*, phenology, timing of reproduction, urbanization

## Abstract

The ecological impact of artificial light at night (ALAN) on phenological events such as reproductive timing is increasingly recognized. In birds, previous experiments under controlled conditions showed that ALAN strongly advances gonadal growth, but effects on egg‐laying date are less clear. In particular, effects of ALAN on timing of egg laying are found to be year‐dependent, suggesting an interaction with climatic conditions such as spring temperature, which is known have strong effects on the phenology of avian breeding. Thus, we hypothesized that ALAN and temperature interact to regulate timing of reproduction in wild birds. Field studies have suggested that sources of ALAN rich in short wavelengths can lead to stronger advances in egg‐laying date. We therefore tested this hypothesis in the Great Tit (*Parus major*), using a replicated experimental set‐up where eight previously unlit forest transects were illuminated with either white, green, or red LED light, or left dark as controls. We measured timing of egg laying for 619 breeding events spread over six consecutive years and obtained temperature data for all sites and years. We detected overall significantly earlier egg‐laying dates in the white and green light vs. the dark treatment, and similar trends for red light. However, there was a strong interannual variability in mean egg‐laying dates in all treatments, which was explained by spring temperature. We did not detect any fitness consequence of the changed timing of egg laying due to ALAN, which suggests that advancing reproduction in response to ALAN might be adaptive.

## Introduction

Since the invention of electric light the extent and radiance of artificial light at night (ALAN) has been growing globally (Kyba et al. 2017). Today more than one‐fifth of the Earth's surface experiences nocturnal anthropogenic illumination and one‐fifth of the human population lives in areas where the Milky Way cannot be seen with the naked eye (Falchi et al. 2016, Kyba et al. 2017). This unprecedented modification of the natural nocturnal environment has sparked interest and concern among astronomers, biomedical scientists, and ecologists alike. In particular, in the last decade, a large amount of literature on the biomedical and ecological effects of ALAN has been accumulating (Rich and Longcore 2006, Wyse et al. 2011, Dominoni et al. 2016). This has been paralleled by an increased attention to the design, development, and implementation of lighting technologies that are able to reduce such effects, including the use of LED lights as they allow for a more flexible tuning of the spectral properties of illumination (Gallaway et al. 2010, Gaston et al. 2012, 2014, Souman et al. 2018). Indeed, many of the known effects of light pollution are spectral dependent (Longcore et al. 2015, Gaston et al. 2017, Spoelstra et al. 2017, Ouyang et al. 2018). In particular, broad‐spectrum lights rich in short wavelengths (i.e., blue light) have been shown to affect several behavioral and physiological responses of animals (van Langevelde et al. 2011, Ouyang et al. 2015, Spoelstra et al. 2015, Bruening et al. 2016).

An obvious reason why light pollution may alter several aspects of the behavior and ecology of wild species is the fact that organisms have evolved under predictable cycles of light and darkness dictated by the sun. Day length is indeed a key driver of daily rhythms of activity, sleep, body temperature, hormone secretion, and gene expression (Foster and Kreitzmann 2004, Roenneberg et al. 2007, Wright et al. 2013, Azzi et al. 2014, Welbers et al. 2017). Similarly, annual changes in photoperiod modulate seasonal reproduction, molt, migration, immune function, and metabolic rate (Gwinner 1987, Nelson and Demas 1997, Bradshaw and Holzapfel 2007, Hut 2011, Helm et al. 2013). Thus, it comes as no surprise that most of the reported effects of ALAN are related to changes in the biological timing of the organisms studied so far. ALAN may affect the perceived photoperiod (Dominoni and Partecke 2015), thus leading to changes in the temporal behavior and physiology of organisms (de Jong et al. 2016, Raap et al. 2016, Botha et al. 2017, Capilla‐Lasheras et al. 2017, Batra et al. 2019, Ulgezen et al. 2019), which in turn could affect fitness (Michael et al. 2003, Yerushalmi and Green 2009, Spoelstra et al. 2016).

In this study, we focus on the effects of light pollution on phenology, and specifically on timing of reproductive decisions. Phenological shifts due to ALAN have been reported in several species, from plants (Bennie et al. 2016, Ffrench‐Constant et al. 2016), to insects (van Geffen et al. 2014), to fish (Bruening et al. 2011), birds (Dominoni et al. 2013, 2018, Da Silva et al. 2015), and mammals (Robert et al. 2015). In species that use long day lengths to time reproductive decisions, such as birds and most tree species, ALAN usually advances reproduction (Kempenaers et al. 2010, Dominoni et al. 2013, Ffrench‐Constant et al. 2016). In short‐day breeders, for instance perch and wallabies, light pollution has been found to delay reproduction (Robert et al. 2015, Bruening et al. 2016). In other cases, for instance some particular plant species and in moths, exposure to ALAN was shown to inhibit reproduction (van Geffen et al. 2014, Bennie et al. 2016). Importantly, most of these phenological effects are spectrum dependent, with broad‐spectrum white light rich in blue light having a stronger impact than light sources dominated by longer wavelengths (van Geffen et al. 2014, de Jong et al. 2015, Bruening et al. 2016).

We have previously shown that in a passerine bird species, the Great Tit (*Parus major*), ALAN can affect both the timing of gonadal maturation (Dominoni et al. 2018) as well as that of the ultimate reproductive decision, egg laying (de Jong et al. 2015). However, the effect on laying dates was found only in one of the two years of our initial study, which was conducted in the field using an experimental set‐up (de Jong et al. 2015). In the first year of the study, 2013, the average time of egg laying in the populations we monitored occurred in mid‐spring (beginning of May). In this year, birds breeding in forest transects experimentally exposed to green and white LED light at night significantly advanced egg laying of 4.3 and 3.8 d compared to the dark control, respectively, while no effect was found for red LED light. In the second year of the study, 2014, average reproductive timing occurred one month earlier than in 2013 (beginning of April). In this year, no effect of any color of ALAN was found on timing of egg laying. Thus, the effects of ALAN on egg‐laying behavior in Great Tits can show considerable annual variation. A key question is what may modulate such variation.

The control of gonadal growth in avian species inhabiting temperate regions is well understood (Dawson et al. 2001). At these latitudes, birds need to grow their gonads every year in advance of the breeding season. Gonadal growth is a long and an energetically expensive process. It takes several weeks for both males and females to grow their testes and ovaries and become ready to produce functional sperm and eggs. Consequently, gonadal growth needs to start much in advance of the predicted egg‐laying time. Photoperiod is extremely predictable as it shows little or no year‐to‐year variation. Thus, birds use the increasing day length in late winter and early spring as a proximate cue to trigger gonadal growth (Dawson et al. 2001). The timing of the initiation of gonadal growth in females has a strong genetic component, with a heritability of 0.73 (Schaper et al. 2013). The duration of gonadal growth shows more variation, probably because the time it takes to grow the gonads can depend on several factors, including the intrinsic energetic state of an animal but also supplementary cues such as temperature and food availability (Schaper et al. 2013). Once gonadal growth is completed, the exact time of egg laying is then modulated by supplementary cues such as temperature, food availability, social stimuli and weather conditions (Dawson et al. 2001), although photoperiod can also influence egg‐laying behavior (Lambrechts and Perret 2000).

Several studies have previously demonstrated that day length and temperature may interact to influence egg‐laying behavior. For instance, Lambrechts and Perret (2000) exposed captive Blue Tits (*Cyanistes caeruleus*) originating from three different populations in Southern France that breed at different times in the wild to the same long day artificial photoperiod treatment, simulating day length of late springs. The non‐photoperiod factors responsible for the differences in breeding time in the wild (temperature and food availability) were overridden by the long photoperiod in captivity. The authors suggested that the relative importance of photoperiodic vs. non‐photoperiodic factors may change as the season progresses (Lambrechts and Perret 2000). The presence of artificial light at night may cause birds to perceive a longer photoperiod, and hence affect the birds’ perception of the time of the year (Titulaer et al. 2012). Because days are longer late in the season, ALAN could “mislead” birds to speed up the reproductive cycle to reduce the known fitness costs associated with a late reproductive attempt (Verboven and Visser 2006). Evidence for a stronger effect of photoperiod relative to non‐photoperiodic cues later in the season also comes from a previous study on Great Tits (Gienapp et al. 2005). The study presented a proportional hazard model to describe and predict variation in the timing of egg laying based on a set of environmental variables. As expected, egg‐laying dates of Great Tits were influenced by spring temperature, mainly via a wide temperature window (i.e., how generally cold or warm a spring is), but to some extent also by short‐term temperature fluctuations. Interestingly, the strength of the effect of temperature was influenced by day length. Specifically, high temperatures early in the spring (under short day length) resulted in earlier egg‐laying dates than the same temperatures under long day length, as expected. This means that a period of warm weather early in spring will, on average, induce more individuals to start egg laying than warm weather in late spring. Conversely, the delaying effect of cold weather is relatively stronger in late spring because late in the season day length became a stronger predictor of egg‐laying date.

The main aim of our study is to understand how between‐year variation in spring temperature interacts with artificial light at night to modulate timing of reproduction in Great Tits. We hypothesized that in cold, but not in warm, springs, Great Tits advance the timing of egg laying in response to ALAN. In order to test our hypothesis, we used 6 yr of data from wild Great Tits breeding in eight different forest sites across the Netherlands. At each of these sites, four different transects were created, each of them experimentally illuminated with artificial light at night of a specific wavelength (white, green, red light) or left dark as control (Spoelstra et al. 2015). Since the photoperiodic response of birds is regulated by hypothalamic photoreceptors and these are most sensitive to wavelengths around 490 nm (Foster et al. 1985, Davies et al. 2012), we would expect broad‐spectrum white light as well as green light to have the stronger effects on reproductive timing, as we have previously shown (de Jong et al. 2015). However, previous research has shown that the light transmission property of the skull is such that red light penetrates much better, which may effectively make the photoperiodic response to red light stronger (Malik et al. 2004, Lewis and Morris 2005). Thus, more research is needed in the wild to assert to which light spectra the avian photoperiodic response is more sensitive.

To accurately quantify potential interactive effects between spring temperature and ALAN, we first conducted a climate window analysis to identify the best temporal window in spring when temperature should have the largest effect on the timing of egg laying. Then, we used average spring temperature data within this temporal window, for each year of the study, to test whether the effect of ALAN on egg‐laying dates depended on spring temperature. Lastly, we tested whether the phenological shifts due to light pollution came with fitness consequences or not. In our previous study, we did not find any effect of ALAN on measures of reproductive success (de Jong et al. 2015). This could have been because the degree of phenological advancement due to ALAN was relatively small, or because of limited sample size (only 2 yr of data). Alternatively, it might also be possible that ALAN could cause the phenological shifts in both birds and prey at the same rate, keeping their phenologies matched. Indeed, previous work suggested that short wavelengths of ALAN advance the time of caterpillar emergence in *Mamestra brassicae* (van Geffen et al. 2014). Although our primary focus was in the costs and benefits of ALAN‐induced early breeding, we were also interested in examining whether ALAN might impact fitness independent of timing of laying.

## Materials and Methods

### Experimental set‐up

In 2011, we established eight sites each with 36 Great Tit nest boxes as part of a larger study on artificial lights’ effect on wildlife (Spoelstra et al. 2015). We placed these study sites in eight previously unlit (dark) natural areas in the Netherlands. Each site consists of four different transects with nine nest boxes (total of 36 boxes per site) with an entrance hole diameter of 32 mm. We assigned each transect within a site was to one of four different light treatments: green, white, and red LED lights or a dark control. We mounted lights on five light posts per transect (4 m tall) and, on the control transects, we installed posts without lights. We installed nest boxes at different distances to the closest light post (de Jong et al. 2015; Appendix S1: Fig. S1). All of the lamps emit full‐spectrum light, as the different colors only represent differences in the level of emission of certain colors: green lamps have an increase of blue light emission and reduction of red and, in the red lamps, red is increased and blue reduced (Spoelstra et al. 2015). We set the light intensity to 7.6 ± 1.2 (mean ± SE) lux at ground level directly under the light post, which is similar to light levels of roads in Northern Europe. We placed the transects perpendicularly in the forest edge. Scots pine (*Pinus sylvestris*) or Douglas fir (*Pseudotsuga menziesii*) dominates the tree cover of each site, except for the site Voorstonden where Oak dominates (*Quercus ruber*). We automatically programmed all light posts to turn on at sunset and turn off at sunrise, throughout the entire year. For a more detailed explanation of the experimental set‐up, we refer to our previous work (Spoelstra et al. 2015).

### Bird data collection

We recorded the breeding behavior of the Great Tits each year from 2013 to 2018 for all sites except Voorstonden, where we could only collect data from 2014 to 2017. We recorded egg‐laying dates by weekly checks of all nest boxes throughout the breeding season, with checks starting in the end of March and ending in late June or early July. When we observed eggs in nests, we back‐calculated the laying date for the first egg by counting the number of eggs, as Great Tits lay one egg per day. We also recorded the number of hatchlings and the number of fledglings and use these data to calculate hatching success (number of hatchlings/clutch size) and fledging success (number of fledglings/number of hatchlings). In this study, we only used data from first broods, thus excluding second and replacement broods.

### Temperature data

We acquired temperature data through the European Climate Assessment and Dataset (ECAD) database (Cornes et al. 2018). We extracted average daily temperature data from 1 January 2013 to 30 June 2018 with a resolution of a 0.25° × 0.25° grid for each of the eight sites. To validate the interpolated temperature data, we placed two temperature loggers in each of the eight sites during the breeding season of 2018. We used iButton Thermochron 8K (accuracy of ±0.0625°C; Maxim Integrated Products, San Jose, CA, USA). We placed the iButtons on the trunk of the tree for two representative nest boxes in each site, 1.5 m above the ground, facing north. The iButtons logged ambient temperature every 15 minutes, starting on 3 April 2018 at 00:00 and ending on 3 June 2018 at 00:00. Temperature data collected from loggers and ECAD database were highly correlated (Pearson coefficient = 0.92, *P* < 0.001; Appendix S1: Fig. S3).

### Climate window analysis

We used the R package *ClimWin* to identify the time window where the ambient temperature will have the largest effect on the Great Tit egg‐laying dates, (Bailey and van de Pol 2016, van de Pol et al. 2016). *ClimWin* uses a sliding window approach on biological data to find the time period that will have the strongest effect on a biological variable. We used weather data from the ECAD database together with breeding data from all 6 yr of the study. We set the boundaries for the sliding window analysis to 1 January to 31 May. The baseline model structure that we used for model testing was a linear mixed model (LMM) with a Gaussian error distribution. We included egg‐laying date as response variable, site as a random factor and temperature as fixed effect. We set the analysis to find an absolute critical time window where ambient temperature will have the strongest effect on the egg‐laying date of the Great Tit populations located at our eight sites.

The *slidingwin* function, the main function of *ClimWin*, compares all possible climate windows in the data set using values of AIC_c_. The results are thus susceptible for possible overfitting, as a climate window could be found by chance rather than based on biological importance. To account for this, we used the *randwin* function, which randomizes the data and reruns the *slidingwin* function so that it is possible to determine how likely the results are to be found by chance. Our data was processed by *randwin* with 250 iterations before the *slidingwin* analysis was performed.

Because the sliding window approach works best when 10 or more years of data are available (Bailey and van de Pol 2016, van de Pol et al. 2016), and we only have 6 yr of data, we may be at risk of overfitting in a relatively small sample size. Thus, we also conservatively run our statistical model (see details in the statistical analyses section) using an already published window (16 March–20 April; Visser et al. 2006). This climate window was calculated using long‐term data (50 yr) obtained from the Great Tit population breeding in the Hoge Veluwe, a National Park very close to most of our sites. We then compared the results of the two approaches.

### Statistical analyses

We ran all analyses in the statistical environment R (R Development Core Team 2015) with a significance level of α = 0.05.

We performed a linear model between the recorded temperature data by the iButtons and the acquired weather data from the database to control the validity of the interpolated temperature data from the ECAD.

We first tested for difference in laying dates between treatments using a (LMM). We included the interaction between treatment and distance to the nearest lamp post because we expected the effect of light to decrease with light intensity (see de Jong et al. 2015). We modeled site and year were as random factors to account for between‐site and between‐year variation (Appendix S1: Table S1).

To test whether the egg‐laying dates in the light treatments depended on the average egg‐laying date of a specific spring, we used an LMM modeling the annual mean laying date of the light treatments for each site as response variable and the respective, site‐specific annual mean laying date for the dark treatment as an explanatory variable. As we were primarily interested in whether the slope of such relationship would be significantly different from 1, we also included an offset in the model (see Appendix S1: Table S2, for full model specifications). In this model, we included site and year as random effects. We also used an alternative way to test whether the slope of the relationship was different from 1 or not, by calculating the confidence intervals of the explanatory variable (annual mean egg‐laying date in dark treatment) from a model without the offset, and assess whether or not these intervals included 1. We did this using the R function *confint*.

Next, we tested if the mean spring temperature significantly predicted the deviation in average egg‐laying dates between a light treatment and the control. Because we obtained mean spring temperature at the site level for each year, it would have been pointless to use this variable and relate it to nest‐box‐level laying dates. Rather, we first calculated site‐level annual means of deviation in laying dates between the three treatments and the dark control. Then, we used a LMM fitting this variable as response, and the light treatment in interaction with the mean spring temperature (calculated using the temporal window defined by *climwin*) as explanatory variables. We included site as random effect (Appendix S1: Table S3). We repeated the same model calculating the mean spring temperature data from the already published window we have mentioned above (Visser et al. 2006).

The final step of our analyses was to assess if any change in timing of reproduction due to the experimental exposure to light had fitness consequences. To this end, we used the same site‐level annual means of deviation in laying dates between the three treatments and the dark control, and we related this to the annual mean of key fitness traits for each treatment in each site. Specifically, we used the deviation in egg‐laying dates, in interaction with light treatment, as explanatory variable in three different models, modeling as response variables the probability of hatching failure (0, at least one chick hatched; 1, no eggs hatched), the total number of hatchlings produced and the total number of fledglings produced. We analyzed the probability of hatching failure using a binomial GLMMs, while we analyzed the number of hatchlings and fledglings with Gaussian LMMs. In all these fitness models, we included clutch size (also averaged per treatment, per site, per year) as a covariate and site as random effect (Appendix S1: Table S4).

We checked the assumptions for using linear models (normality and homogeneity of residuals) and they were met in all models. We computed *P* values using likelihood ratio tests. If we found a significant effect of the variable treatment, we computed post‐hoc tests (contrasts) with the function *emmeans* in the R package *emmeans*. We adjusted *P* values for the contrast between two treatment groups using the Tukey's test.

## Results

### Effects of ALAN on timing of egg laying

**Figure 1:**
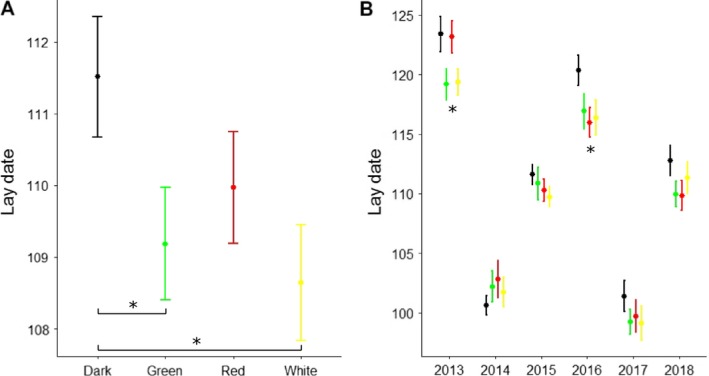
Variation in egg‐laying date depends on both light treatment and year of study. (A) Female Great Tits breeding in the white and green light treatments (indicated by bars and asterisks) laid earlier than their conspecifics in the control dark sites (data pooled across years). (B) Average egg‐laying dates per treatment were strongly affected by the study year (significant [*P* ≤ 0.05] year‐specific effects depicted by asterisks). Data are shown as mean ± SE. Egg‐laying dates are expressed as number of days from 1 January.

We detected a significant effect of treatment on egg‐laying dates (*P* = 0.015 and Fig. 1A; Appendix S1: Table S1). Females in the white and green light transects laid their eggs on average 2.1 and 1.9 d earlier than females in the dark transects, respectively, and these differences were significant (Tukey‐adjusted post‐hoc test, *P* = 0.020 and *P* = 0.045; Appendix S1: Table S1). Females in the red‐light treatment also laid earlier on average than birds in the dark control areas, but not significantly so (estimate = −1.5, *P* = 0.160). The effect of the light treatments on timing of egg laying did not depend on the distance to the nearest lamp post (treatment × distance interaction, *P* = 0.76; Appendix S1: Table S1). Interannual differences in egg‐laying dates were strong (significance of random effect year, *P* < 0.001, Fig. 1; Appendix S1: Table S1). Individual egg‐laying dates ranged from 29 March to 20 May across all years. When we tested our model on each year separately, as a post‐hoc test, we found a significant effect of treatment on egg‐laying dates only in 2013 (*P* = 0.037) and 2016 (*P* = 0.049).

**Figure 2:**
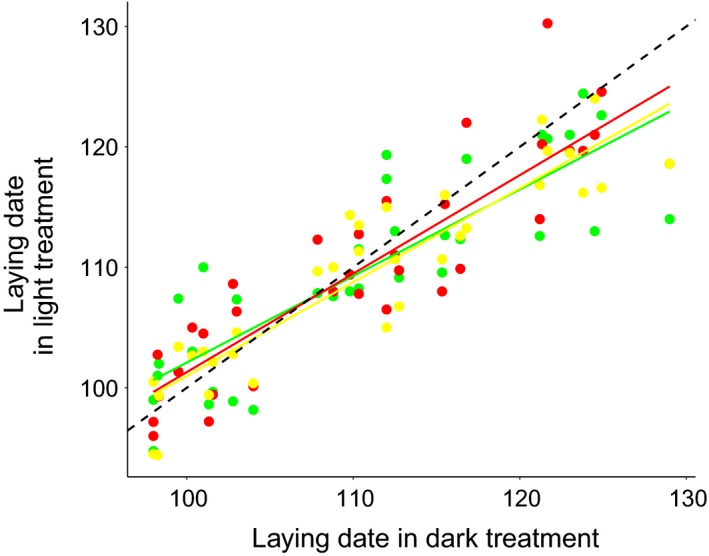
Average egg‐laying date in one year predicts the effect of light treatment on egg‐laying dates. In late years (right end of the graph), females in the light treatments laid on average significantly earlier than females in the control dark group. Conversely, in warm years (left end of the graph), average egg‐laying date in all population was early and light pollution did not advance egg‐laying dates compared to the dark group. Egg‐laying dates are expressed as number days from 1 January. Colour lines are regression liens from the model for each treatment, dashed line is the 1:1 reference line.

Egg‐laying dates in the light transects in a given year were related to egg‐laying dates in the dark control transects in the same year. However, the slope of such relationship was significantly smaller than 1 (*P* = 0.019, Fig. 2; Appendix S1: Table S2): when females in the dark treatment laid late in spring, females in the light treatments laid also late but to a lesser extent. This result was also confirmed when we ran R function *confint* on the same model without the offset, as the confidence intervals of the explanatory variable did not contain 0. Such a relationship was not influenced by the specific treatment (interaction light treatment × egg‐laying date in dark treatment, *P* = 0.52; Appendix S1: Table S2), though the effects were stronger for the white and green treatment compared to the red one (green, estimate =−1.2; red, estimate = −0.7; white: estimate = −1.6).

### Climate window analysis

The climate window analysis indicated that average egg‐laying date was best correlated with the daily mean temperature between 27 March and 11 April (*P* < 0.001, corrected for multiple testing). The climate window range for the 95% confidence interval obtained from 250 randomizations of the climate model fell between these two dates (Appendix S1: Fig. S2) and thus suggests this is the best fitting period for egg‐laying dates. This 14‐d window is considerably shorter than what was previously found using a much longer data set (34 d; Visser et al. 2006).

### Relationship between temperature and egg‐laying behavior

**Figure 3:**
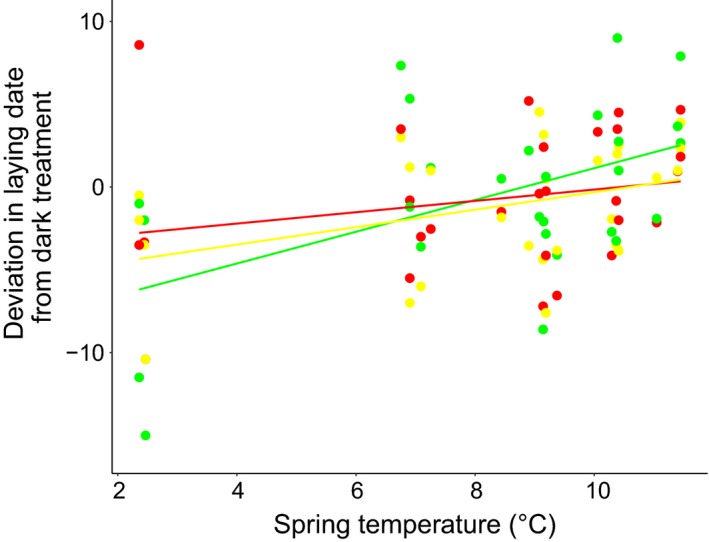
Spring temperature modulates the effect of light treatment on the timing of egg laying. The deviation in average egg‐laying dates (expressed as number of days) between the light and the dark control treatments was stronger in cold springs compared to warm years. This relationship did not depend on the light treatment. Each dot in the figure represents mean egg‐laying dates for a single site in a single year. Lines represent model predictions.

We used the average temperature data for the best fitting climate window of each year to examine how variation in spring temperature affected the relationship between light treatments and egg‐laying behavior. Spring temperature was related to the deviation in egg‐laying dates (*P* < 0.001; Fig. 3; Appendix S1: Table S3). In other words, when spring temperature was low female Great Tits advanced egg‐laying date compared to females in the dark control transects. The advancement was 0.56 d/°C on average across light treatments. Despite the data suggesting that the relationship between temperature and deviation in egg‐laying dates was stronger in the white and green treatment compared to the red one, the interaction treatment × deviation was not significant (*P* = 0.363). When we reran this model using the already‐published climate window (Visser et al. 2006), the results did not change (Appendix S1: Table S3).

### Fitness consequences of changes in timing of egg laying due to ALAN

**Figure 4:**
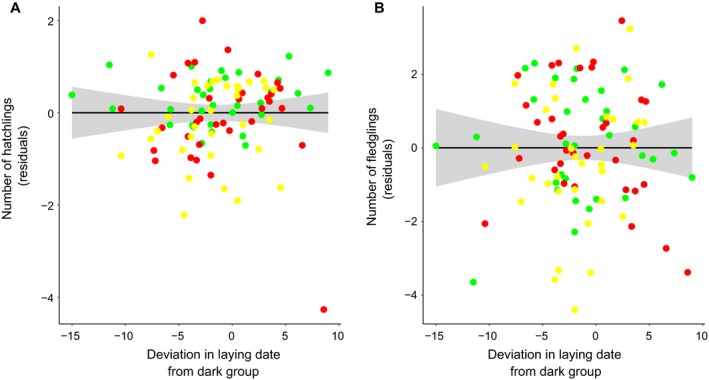
Shifting phenology due to artificial light at night does not have fitness consequences. (A) The difference in egg‐laying date between the light treatments and the control dark group (expressed as number of days) did not predict the number of hatchlings produced. Similarly, (B) the difference in egg‐laying date between the light treatments and the control dark group did not affect the number of fledglings produced. Each data point in the figure represents the residuals of a model containing the number of hatchlings or fledglings as response variable and the mean deviation in egg‐laying date per treatment per year as explanatory variable. Clutch size was also included in the model as a covariate. Lines and shaded areas represent model predictions ±95% confidence intervals.

Fitness traits were not affected by the deviation in egg‐laying dates related to ALAN (Fig. 4; Appendix S1: Table S4). The probability of a brood failure was not influenced by the difference in egg‐laying dates between the light treatments and the control dark group (*P* = 0.74). The number of hatchlings (*P* = 0.52) and fledglings (*P* = 0.12) were also unaffected by the difference in egg‐laying dates between the light treatments and the dark control (Fig. 4; Appendix S1: Table S4). In all these models, treatment was never a significant predictor of reproductive traits (*P* > 0.09 in all cases; Appendix S1: Table S4).

## Discussion

Light pollution is increasing in radiance and extent worldwide, and is thought to pose a considerable risk to wild populations. In particular, artificial light at night has been shown to alter the timing of reproductive events. However, previous studies have suggested that such alteration of reproductive timing does not occur every year, indicating that climatic conditions might modulate the effect of light pollution. Here, we have experimentally shown that breeding under artificial light at night is associated with a temperature dependent advancement of the timing of egg laying of female Great Tits. This effect was particularly apparent in late, cold springs, but it was not found in warm, early springs. We did not find any interaction between treatment and the distance of a nest box to the closest light post. We suggest two potential explanations for this lack of distance effect. First, Great Tits in the pre‐laying period may not be so tied to their nest box as they are during incubation and chick‐rearing. Thus, birds may move considerably within the same transect area, possibly also roosting in different places every night, as previously suggested (Ouyang et al. 2017). Second, the whole effect of light pollution advancing egg‐laying dates may work indirectly via an increase in the availability of caterpillars in the green and white light transect, as we have recently shown (Welbers et al. 2017). All pairs within the same transect will likely make use of this additional food supply, independently of how distant their nest box is from the closest lamp post.

We next sought to mechanistically explain such a year‐dependent effect of artificial light by using spring temperature data from our study sites. We first identified the temporal window during spring when daily mean temperatures were best correlated to egg‐laying dates in our populations. The identified window falls within the best fitting period found for Great Tits breeding in the Hoge Veluwe (Visser et al. 2006), an area very close to most of our study sites. Using temperature data from this window, we then showed that cold spring temperatures are related to a stronger deviation of egg‐laying dates between the light treatments and the control dark areas, suggesting that spring temperature modulates the effect of artificial light at night on timing of egg laying in female Great Tits. Our results are in line with the previous evidence to which we referred in the introduction, which suggested that sensitivity to temperature varied with photoperiod (Gienapp et al. 2005). This supports the hypothesis that artificial light at night can interact with temperature in affecting laying dates of wild songbirds. However, much of the interactive effect of temperature and light pollution found in our data set is explained by a single year, 2013 (Fig. 3). Thus, more years will be needed to confirm this result.

The effects of ALAN on egg‐laying date were more pronounced for white and green light. This result, albeit confirming what shown in our first study (de Jong et al. 2015), also contrasts with evidence from previous work. Indeed, several studies that have examined spectrum‐dependent effects of photoperiod on timing of reproduction have suggested that red light is more stimulatory to reproductive development than blue or green light (for poultry work, see Lewis and Morris 2005; for songbird work see Malik et al. 2004). This is expected as red light penetrates the skull more (Lewis and Morris 2005) and thus is more capable of reaching the hypothalamic photoreceptors that are the main structures regulating the activation of the gonadal axis in birds (Davies et al. 2012). However, in these experiments, the authors focused only on gonadal development as a measure of timing of reproduction. In poultry research, early studies have failed to reveal a strong effect of light spectrum on egg‐laying behavior (Lewis and Morris 2005), but recent work has shown that red light can stimulate earlier and greater egg production (Yang et al. 2016). However, these poultry studies were not really designed to manipulate light at night, but rather use a long photoperiod to induce reproductive activation and as such their results are not directly comparable to light at night studies. We speculate that the effect of an extended photoperiod late in the spring on the timing of egg laying has more to do with day length detection through the retina rather than with the deep‐brain photoperiodic response. Since retinal photoreceptors are more sensitive to short wavelengths of the visible spectrum (Brandstätter 2003, Cassone 2014, Surbhi and Kumar 2015), white and green light should have a stronger effect on egg‐laying behavior. Alternatively, as mentioned above, the effects of artificial light at night on egg‐laying data may come indirectly through effects on the food resources. Distinguishing between direct vs. indirect effects of light pollution on bird phenology is an outstanding question that future research should investigate. This generally applies to many of the known behavioral effects of light at night on birds, as in most cases the mechanism behind these effects is unknown.

Despite the significant advance in egg‐laying dates due to particularly white and green light at night, we did not detect any fitness consequences of such phenological shift, neither on hatching nor on fledging numbers and success. The lack of fitness effects might be expected if the insects that Great Tits rely on to feed their young, caterpillars, also shift in response to light pollution. In fact, the development of Lepidoptera eggs and larvae is also sensitive to both temperature and photoperiod, although the relative importance of each depends on the species examined (Cox 1979, Fantinou et al. 1996, Nabeta et al. 2005, Tauber et al. 2015, Salis et al. 2018). A previous study has shown that both green and white light at night advances caterpillar emergence from the pupa stage (van Geffen et al. 2014). Thus, it is conceivable to imagine that the timing of caterpillar phenology also undergoes a similar shift in response to ALAN compared to Great Tits, reducing the likelihood of a mismatch to happen. We have previously published caterpillar abundance and phenology data from our study sites (Welbers et al. 2017). While we have shown that green and white light strongly increased the availability of caterpillars, we have detected no effect of any light color on the timing of the spring caterpillar peak. However, for this analysis we crucially missed the year 2013, which is the year where we observed the strongest advance in birds’ laying dates. Moreover, most caterpillar samples that we were able to collect came only from one site, the richest in deciduous trees. In other sites dominated by evergreen trees contained caterpillars were far less abundant. Thus, with the data that we currently have we cannot properly test whether caterpillars shift their peak emergence date to the same extent of birds in response to light pollution. However, a previous study has shown that timing of bud burst, a good predictor of caterpillar peak date (van Asch and Visser 2006), is significantly advanced by light pollution across the UK (Ffrench‐Constant et al. 2016). We thus anticipate that future research should focus on assessing whether trophic interactions across the plant–caterpillar–Great Tit chain are affected by artificial light at night. In addition, we also did not find any effect of the light treatments on breeding output, independently of the timing of egg laying. This suggests that light pollution likely does not affect the reproductive fitness of Great Tits, neither via direct effects (for instance increasing stress levels that could impair reproduction) nor via indirect effects (ALAN‐induced change in caterpillar abundance and phenology).

Our study has some clear limitations that future work should take into account. First, our claim that the effect of light pollution on egg‐laying dates depends on spring temperature mostly relies on the very late seasons of 2013 and 2016. In this respect, our study spans only a period of six years, while the most important avian phenological studies to date can rely on several decades of data (Crick et al. 1997, Both and Visser 2001, Charmantier et al. 2008, Reed et al. 2013, Roberts et al. 2015). To conclusively demonstrate this interactive effect more years of data collection seem necessary. An alternative approach could be to use egg‐laying data from citizen science programs such as the nest record schemes of the UK, Netherlands, and USA (Crick et al. 2009, Møller and Fiedler 2010). These data sets could be integrated with temperature time series as well as light pollution maps, which are now available globally at a very fine spatial resolution (Falchi et al. 2016). A recent study attempted such an analysis and found no evidence for an interaction between ALAN and how late a spring was (de Jong et al. 2018). However, this study did not specifically include temperature as covariate in the models and used egg‐laying dates from nest records obtained in areas with limited level of light pollution. Another limitation of our study is that we do not have any control on the settlement decisions of the birds we studied. Individuals with different sensitivity/tolerance to light at night might settle in different light treatments, thereby biasing our results. Still, we believe that our field set‐up is currently the best approach available that allows to test our hypothesis in a realistic setting using an experimental design. An alternative, a fully experimental approach would be to design a captive study where exposure to artificial light at night is combined with different spring temperature treatments.

Our study suggests that the known effects of both artificial light at night and ambient spring temperature on the timing of avian reproduction can interact with each other. We believe that our results are crucial in improving our understanding of how phenological events are affected in an era of increasing worldwide urbanization and climate change. In fact, our results suggest that temperature may override light pollution in determining timing of breeding when temperatures rise. This is interesting in the face of global warming, as one interesting question could be whether temperature or light is the bigger threat to bird reproduction. Our work shows that when pre‐breeding temperatures increase, there is basically no effect of light on reproduction of passerine birds, suggesting that rise in temperature may be a bigger threat to avian seasonal timing than light pollution.

## Data Availability

Data are available from the Dryad Digital Repository: https://doi.org/10.5061/dryad.8931zcrm0
