# Supplementary material for: Artificial light at night, in interaction with spring temperature, modulates timing of reproduction in a passerine bird
Source: Ecol Appl. 2020 Jan 13;30(3):e02062. doi: 10.1002/eap.2062 (PMC7187248; doi:10.1002/eap.2062)
Supplement: Supplementary file 1 [file EAP-30-e02062-s001.pdf]

Supporting Information. Davide M. Dominoni, Johan Kjellberg Jensen, Maaike de Jong, Marcel E. Visser, and Kamiel Spoelstra. 2020. Artificial light at night, in interaction with spring temperatures, modulates timing of reproduction in a passerine bird. *Ecological Applications*.

Appendix S1

Supporting figures

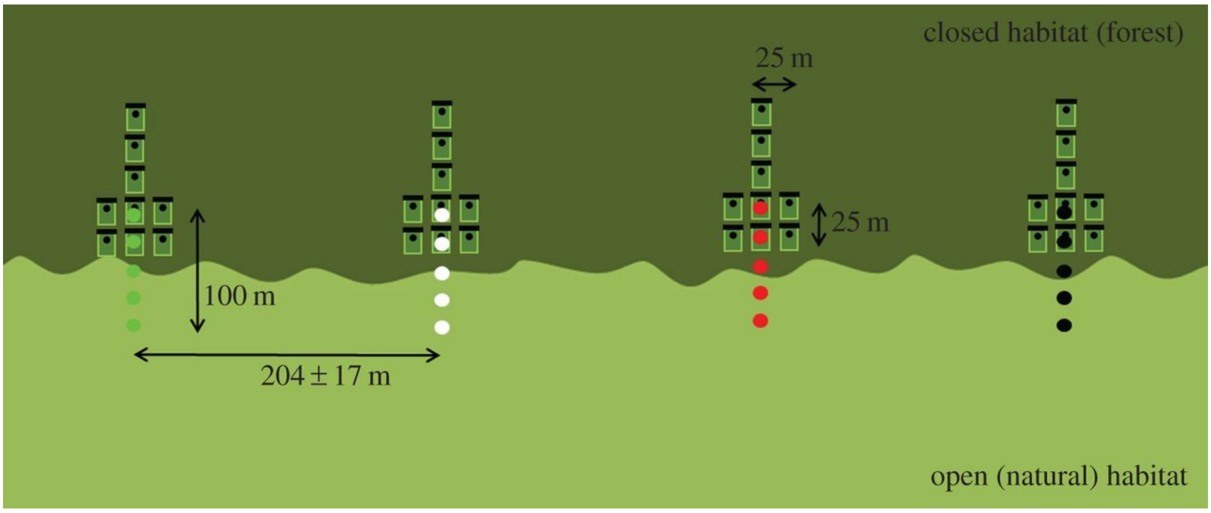

Figure S1. Schematic overview of the set-up of one study site, which is replicated eight times. Five lamp posts are placed in transects perpendicular to the forest edge. Within a site, orientation of transects is constant. Distance between transects is variable and depends on the local situation. Each transect was randomly assigned to one of the four light treatments, here green, white, red and dark, respectively. In each transect, nine nest-boxes were attached to trees at 1.6 m height and at approximately 25 m distance from each other (dependent on the nearest tree). Orientation of the nest-box opening was always towards the forest edge.

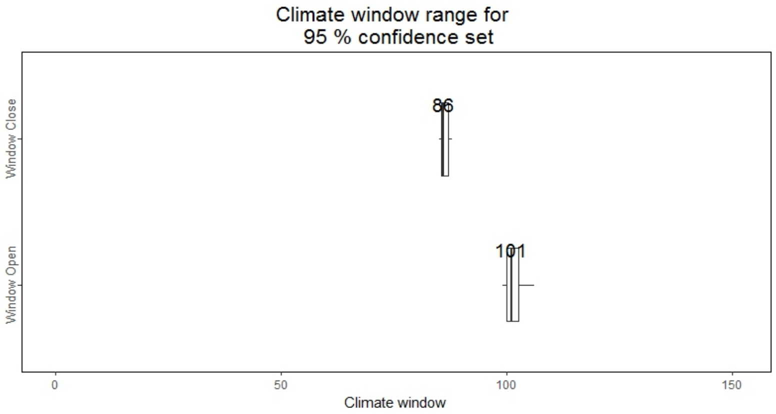

Figure S2. The climate window analysis shows that the best temporal interval of daily mean temperature predicting egg-laying date falls between March 27th and April 11th.

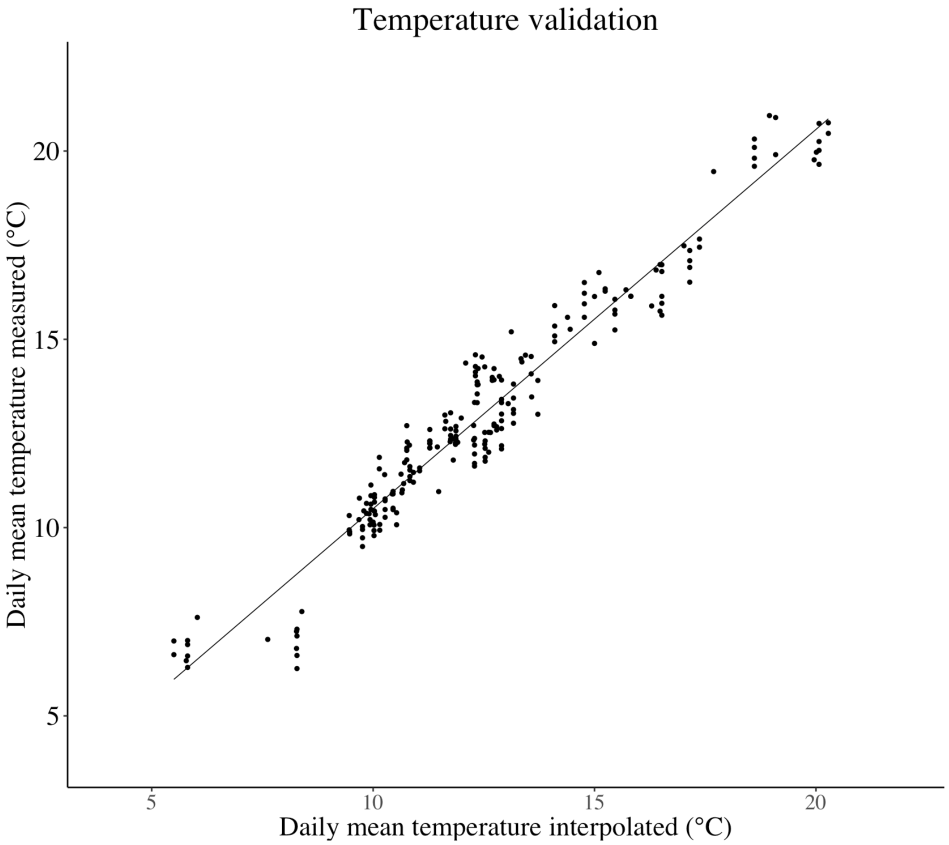

Figure S3. Correlation between temperature data obtained from the ECAD interpolated weather stations dataset (x-axis) and those collected on ground via iButton loggers (y-axis).

Supporting tables

Table S1: Effects of ALAN on egg-laying date (figure 1 in main manuscript). Model is a Gaussian LMM with treatment, distance to nearest lamp post, and their interaction as fixed effects, while site and year where included as random effects. This analysis was conducted at the nest box level. Degrees of freedom, F values and P values are based on likelihood ratio (chi) square tests. Estimates of fixed effects are based on the output of the *lmer* function in R. The interaction term was not significant and is not shown.

Effect of ALAN on egg-laying date (Gausssian LMM, n = 619)

**Fixed effects**	**Estimate**	**df**	**F**	**P value (χ)**
Treatment		3, 604	3.49	**0.015**
Green	-1.9			
Red	-1.4			
White	-2.1			
Distance	-0.02	1, 606	3.67	0.055

**Random effects:**	**Variance**	**St.Dev**
Site	3.92	1.98
Year	68.01	8.24
Residual	39.4	6.28

Post-hoc test (Tukey's adjusted)

Contrasts:	**Estimate**	**SE**	**d.f.**	**t.ratio**	**p.value**
Dark - Green	1.88	0.72	604.32	2.61	**0.045**
Dark - Red	1.45	0.70	604.23	2.08	0.160
Dark - White	2.11	0.72	604.30	2.91	**0.020**
Green - Red	-0.43	0.71	604.18	-0.61	0.930
Green - White	0.23	0.74	604.24	0.31	0.990
Red - White	0.66	0.72	604.41	0.92	0.796

Table S2: Relationship between egg-laying date in dark treatment and egg-laying date in light treatments (Figure 2 in main manuscript). Model is a Gaussian LMM with egg-laying date in dark treatment as fixed effect, while site and year where included as random effects. This analysis was conducted at the transect level, averaging site-specific egg-laying dates per treatment per year. Degrees of freedom, F values and P values are based on likelihood ratio (chi) square tests. The estimate of the fixed effect is based on the output of the *lmer* function in R. Because we were interested in testing whether the slope of the relationship was different than one (see main text), we included an offset in the model, The model formula was therefore: *mod14<-lmer(LayLight~LayDark+(1|Site)+(1|Year), data=data, offset = LayDark).*

Relationship between egg-laying date in light treatments and dark treatment (Gausssian LMM, n = 99)

**Fixed effects:**	**Estimate**	**d.f.**	**F**	**p-value (χ)**
Egg-laying date in dark treatment	-0.29	1, 3	18.12	**0.019**

**Random effects:**	**Variance**	**St.Dev**
Site	3.84	1.96
Year	1.90	1.38
Residual	10.95	3.31

Table S3: Effects of spring temperature on the deviation in egg-laying date between dark and light treatments (figure 3 in main manuscript). Model is a Gaussian LMM with temperature, treatment and their interaction as fixed effects, site and year as random effects. This analysis was conducted at the transect level, averaging site-specific egg-laying dates per treatment per year. Degrees of freedom, F values and P values are based on likelihood ratio (chi) square tests. Estimates of fixed effects are based on the output of the lmer function in R. The interaction term was not significant and is not shown. The model was run using our climwin-defined window, as well as an already published climate window (see main text). These are shown in panels A and B, respectively.

Effect of temperature on deviation in laying date between dark and light treatments (Gaussian LMM, n = 99)

A: climwin dataset

**Fixed effect:**	**Estimate**	**d.f.**	**F**	**p-value (χ)**
Mean temperature	0.66	1, 74	17.87	**<0.001**
Red treatment	-0.24	2, 73	0.32	0.807
White treatment	-0.72	2, 73	0.32	0.574

**Random effects:**	**Variance**	**St.Dev**
Site	5.44	2.33
Residual	13.55	3.68

B: published window

**Fixed effect:**	**Estimate**	**d.f.**	**F**	**p-value (χ)**
Mean temperature	1.16	1, 74	17.87	**<0.001**
Red treatment	-0.24	2, 73	0.32	0.803
White treatment	-0.72	2, 73	0.32	0.465

**Random effects:**	**Variance**	**St.Dev**
Site	5.72	2.39
Residual	12.99	3.60

Table S4: Models assessing the relationship between the deviation in egg-laying date between dark treatment and light treatments and its consequences of three parameters of reproductive success (Figure 4 in main manuscript). Models contained this deviation variable in interaction with light treatment, as well as clutch size, as fixed effects, and site as random effect. This analysis was conducted at the transect level, averaging site-specific egg-laying dates and clutch sizes per treatment per year. Degrees of freedom, F values and P values are based on likelihood ratio (chi) square tests. Estimates of fixed effects are based on the output of the *lmer* function in R. Non-significant interactions were removed.

Effect of deviation in laying date caused by ALAN on fitness traits

Brood failure (Binomial GLMM, n=101)

	Estimate	d.f.	F	p-value (χ)
Deviation in lay date between light and dark treatment	0.04	1, 94	1.01	0.74
Light Treatment		2, 89	0.69	0.25
Red	-2.49			
Green	-1.3			
Clutch size	1.18	1,94	6.58	**0.010**

Number of hatchlings (Gaussian LMM, n=99)

	Estimate	d.f.	F	p-value (χ)
Deviation in lay date between light and dark treatment	0.01	1, 94	0.41	0.52
Light Treatment		2, 89	2.44	0.09
Red	-0.38			
Green	-0.47			
Clutch size	1.01	1, 94	129.9	**<0.001**

Number of fledglings (Gaussian LMM, n=99)

	Estimate	d.f.	F	p-value (χ)
Deviation in lay date between light and dark treatment	0.06	1, 94	2.33	0.12
Light Treatment		2, 89	1.17	0.31
Red	0.01			
Green	-0.54			
Clutch size	0.53	1, 94	10.19	**0.002**
